# Monthly Reports

**Published:** 1803-09-01

**Authors:** J. Ricards

**Affiliations:** No. 68, Hatton Garden


### MONTHLY REPORTS.

*Cases admitted under the Care of the Surgeon of the Finsbury Dispensary, St. John's Square, Clerkenwell, from July* 10, *to August* 10, 1803.

| | | | |
|---|---|---|---|
| Phlegmone Artuum | 2 | Gonorrhœa | 1 |
| Hernia Humoralis | 1 | Hydrarthus Ischii | 1 |
| Ophthalmia | 1 | Eruptio | 1 |
| Erysipelas | 3 | Tinea | 2 |
| Abscessus Axillæ | 1 | Varices | 2 |
| Mortificationes | 3 | Scrophula | 1 |
| Ulcera Cruris | 13 | Rachitis | 1 |
| Contusiones | 6 | Steatoma | 1 |
| Vulnus | 1 | | |
| Fractura Brachii | 1 | Total | 47 |
| Lues | 5 | | |

Nearly about the present season of the last year, during the most intense heat of the summer, I had occasion to observe that the diseases which created most alarm, and which were extremely frequent, were Gangrenes; the same remark pertains to the present season; during the month which this Report includes, there have been several cases of the kind, two of which have arisen spontaneously, have advanced in the most rapid manner, and have every appearance of a fatal termination.

The trivial nature of the origin of these diseases, and their rapid progress, seem to indicate a very peculiarly languid state of corporeal energy; which, in some measure, may be attributed to the excessive heat of the season, acting on constitutions, perhaps, not previously of the soundest stamina, and on persons rather advanced in life.

The subject of the first of these cases is a woman of 60 years of age, of a delicate habit; some weeks since a small pimple appeared under her right breast, which she rubbed off by accident; the edges inflamed and the centre assumed a gangrenous aspect; this centre very speedily enlarged, spreading in every direction. When I first saw it, it was about as large as a half-crown piece; I directed fermenting cataplasms, and internally cinchona and opium; no interruption to the progress of the disease was, however, produced; the integuments and cellular membrane sloughed; and as it contined to separate in the centre, the mortification proceeded along the circumference; the whole of the right breast sloughed away; and at the present time, the extent of the disease is from the axilla upwards to the

 groin groin downward, and from the centre of the sternum and linea alba forward, nearly to the spine, in a posterior direction.

The other case is in an aged man; the disease proceeded from irritation produced by scratching the foot; gangrene commenced, and destroyed nearly the whole of the foot to the ancle-joint; the progress of the disease has however ceased, and a separation has commenced, but in all probability amputation of the limb will be required, as Nature appears sinking, and unable to support the length of exertion requisite to throw off parts of such small degree of vitality as the tendons and ligaments, which occupy so large a part of the foot.

*No. 68, Hatton Garden.* J. RICARDS.

